# Microdeletion del(22)(q12.2) encompassing the facial development-associated gene, *MN1 *(meningioma 1) in a child with Pierre-Robin sequence (including cleft palate) and neurofibromatosis 2 (NF2): a case report and review of the literature

**DOI:** 10.1186/1471-2350-13-19

**Published:** 2012-03-22

**Authors:** Tom B Davidson, Pedro A Sanchez-Lara, Linda M Randolph, Mark D Krieger, Shi-Qi Wu, Ashok Panigrahy, Hiroyuki Shimada, Anat Erdreich-Epstein

**Affiliations:** 1 Department of Pediatrics and the Saban Research Institute at Children's Hospital Los Angeles, 4650 Sunset Boulevard, Los Angeles, California 90027-6062, USA; 2 Department of Surgery and the Saban Research Institute at Children's Hospital Los Angeles, 4650 Sunset Boulevard, Los Angeles, California 90027-6062, USA; 3 Department of Radiology and the Saban Research Institute at Children's Hospital Los Angeles, 4650 Sunset Boulevard, Los Angeles, California 90027-6062, USA; 4 Department of Pathology and the Saban Research Institute at Children's Hospital Los Angeles, 4650 Sunset Boulevard, Los Angeles, California 90027-6062, USA; 5 The Keck School of Medicine, University of Southern California, 1975 Zonal Avenue, Los Angeles, CA 90089-9034, USA; 6 Division of Hematology/Oncology, Mattel Children's Hospital, David Geffen School of Medicine at UCLA, Los Angeles, CA 90095-1752, USA

**Keywords:** Chromosome 22q12.2, Cleft palate, MN1, NF2, Pierre-Robin sequence

## Abstract

**Background:**

Pierre-Robin sequence (PRS) is defined by micro- and/or retrognathia, glossoptosis and cleft soft palate, either caused by deformational defect or part of a malformation syndrome. Neurofibromatosis type 2 (NF2) is an autosomal dominant syndrome caused by mutations in the *NF2 *gene on chromosome 22q12.2. NF2 is characterized by bilateral vestibular schwannomas, spinal cord schwannomas, meningiomas and ependymomas, and juvenile cataracts. To date, NF2 and PRS have not been described together in the same patient.

**Case presentation:**

We report a female with PRS (micrognathia, cleft palate), microcephaly, ocular hypertelorism, mental retardation and bilateral hearing loss, who at age 15 was also diagnosed with severe NF2 (bilateral cerebellopontine schwannomas and multiple extramedullary/intradural spine tumors). This is the first published report of an individual with both diagnosed PRS and NF2. High resolution karyotype revealed 46, XX, del(22)(q12.1q12.3), FISH confirmed a deletion encompassing *NF2*, and chromosomal microarray identified a 3,693 kb deletion encompassing multiple genes including *NF2 *and *MN1 *(meningioma 1).

Five additional patients with craniofacial dysmorphism and deletion in chromosome 22-adjacent-to or containing *NF2 *were identified in PubMed and the DECIPHER clinical chromosomal database. Their shared chromosomal deletion encompassed *MN1*, *PITPNB *and *TTC28*. *MN1*, initially cloned from a patient with meningioma, is an oncogene in murine hematopoiesis and participates as a fusion gene (*TEL*/*MN1*) in human myeloid leukemias. Interestingly, *Mn1*-haploinsufficient mice have abnormal skull development and secondary cleft palate. Additionally, *Mn1 *regulates maturation and function of calvarial osteoblasts and is an upstream regulator of *Tbx22*, a gene associated with murine and human cleft palate. This suggests that deletion of *MN1 *in the six patients we describe may be causally linked to their cleft palates and/or craniofacial abnormalities.

**Conclusions:**

Thus, our report describes a *NF2*-adjacent chromosome 22q12.2 deletion syndrome and is the first to report association of *MN1 *deletion with abnormal craniofacial development and/or cleft palate in humans.

## Background

Pierre-Robin sequence (PRS) refers to a combination of micrognathia or retrognathia, glossoptosis and respiratory distress, with or without cleft palate, named after the French stomatologist, Pierre Robin [1-5]. The incidence of PRS is estimated at 1 in 8,500-14,000 births and continues to be associated with high morbidity secondary to a compromised airway, feeding difficulties, and speech problems [6-9]. While the clinical presentation is well defined, pathogenesis is heterogeneous and not completely understood. In half or more of patients PRS occurs in isolation [2,4,5,7]. Isolated PRS is often thought to be due to intrauterine fetal constraint where extrinsic physical forces (e.g., oligohydramnios, breech position or abnormal uterine anatomy) inhibit normal mandibular growth. Micrognathia in early fetal development may then cause the tongue to remain between the palatal shelves, thus interfering with palate closure [2,10]. However, this mechanism has been challenged by some [11]. Isolated PRS can also be associated with chromosomal deletions such as 2q24.1-33.3, 4q32-qter, 11q21-23.1, and 17q21-24.3 [6]. Recent reports of isolated PRS have additionally found mutations in highly conserved non-coding elements surrounding *SOX9 *at 17q24 [6,12,13]. Frequently, PRS is associated with additional clinical findings that may or may not constitute part of a recognized syndrome. The most common PRS-associated syndromes include Stickler syndrome, velo-cardio-facial syndrome (VCFS, associated with chromosome 22q11.2 mutations), fetal alcohol syndrome and trisomy 18 [3-5].

Neurofibromatosis type 2 (NF2) is an autosomal dominant disorder that predisposes to multiple neoplastic lesions. Birth incidence is estimated to be between 1 in 25,000 to 1 in 40,000 births [14-18]. The distinctive features of NF2 are eighth cranial nerve schwannomas, which occur bilaterally in over 90% of NF2 patients [14,16,19]. Patients may also develop schwannomas in other locations, particularly the spine, as well as meningiomas, ependymomas, and juvenile cataracts. NF2 is typically caused by truncating mutations (nonsense or frameshift) of the *NF2 *gene on chromosome 22q12.2, which encodes the intracellular membrane-associated tumor suppressor protein, merlin, also called schwannomin [20-23]. While most NF2 patients are diagnosed in their second or third decades of life, cataract, hearing loss, facial weakness, headache or ataxia may occur during childhood.

To date, there have been no published reports of patients diagnosed with both PRS and NF2. Here we provide the first description of clinical association between PRS (including cleft palate) and NF2 in a patient in whom we found a 22q12.2 deletion. Interestingly in addition to *NF2*, this deletion also encompasses *Meningioma1 *(*MN1*), a gene that is important in murine craniofacial development, and whose haploinsufficiency in mice causes skull abnormalities and secondary cleft palate, but which to date, has not been associated with malformations in humans.

## Case presentation

**Figure 1:**
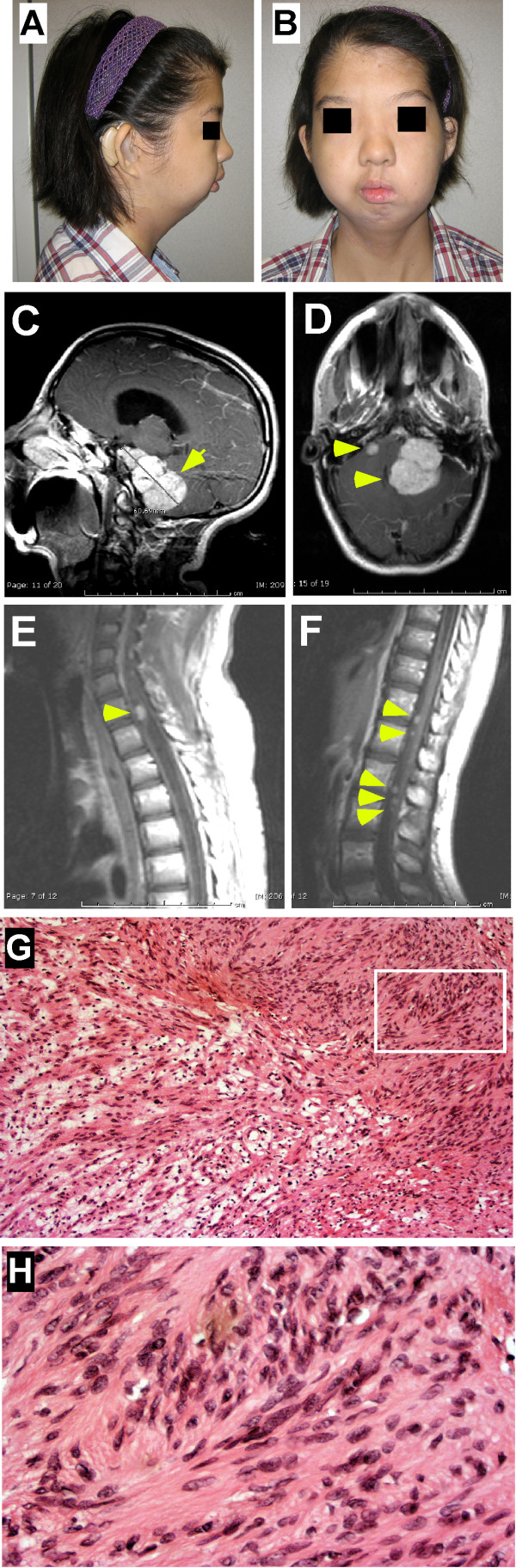
**Patient PRS-NF2, clinical presentation**. **A-B**) PRS-NF2 at age 17 years with severe micrognathia, ocular hypertelorism and microcephaly. Measurements: outer canthal distance 8.3 cm, 25^th ^%ile; inner canthal distance 4 cm, 3 S.D. above the mean; interpupillary distance 5.5 cm, 25^th ^%ile; head circumference 52 cm, 2^nd ^%ile, weight 41.8 kg, 2-1.4 S.D. below the mean; height 156 cm, 10^th^-25^th ^%ile. **C-D**) MRI of brain shows bilateral cerebellopontine angle tumors (left: 3.1 × 4.6 × 6.1 cm, right: 1 × 1.2 × 1.1 cm; arrows) with mass effect by the left tumor on the brain stem, pons and cerebellum, with associated effacement of the 4^th ^ventricle, enlargement of the 3^rd ^and lateral ventricles, enlarged cervical cord and tonsillar herniation. **E-F) **MRI of spine shows holocord syrinx with large neurofibroma at C7-T1 (E), causing cord compression and canal stenosis. Some of the other extramedullary intradural neurofibromas described in the text are also shown. **G-H**) Histological analysis of the resected left cerebellopontine angle tumor shows spindled nuclei with dense to finely granular chromatin and cells with long tapering cytoplasmic processes with inconspicuous outer membranes. Panel G demonstrates both short fascicular bundles with dense Antoni type A area (right upper corner) and loose hypocellular Antoni type B area (middle to left lower corner) characteristic of schwannomas (H&E, G-100×). Panel H is 400 × magnification of the inset from panel G).

Our patient (PRS-NF2) was born to healthy non-consanguineous parents (mother 39 years old) at week 36 by emergency cesarean section due to premature contractions and early decelerations. Pregnancy was otherwise uncomplicated, without report of oligohydramnios or breech presentation. Cleft palate, micrognathia, microcephaly and ocular hypertelorism were noted at birth and the patient suffered multiple apneic episodes. For the first year she was fed by nasogastric tube due to poor weight gain. Motor development was delayed (rolled at 8 months, sat at 10 months, walked at 18 months), and she was later diagnosed with mental retardation. From preschool age she had bilateral severe conductive hearing loss requiring hearing aids that was attributed to bony changes associated with her congenital dysmorphic features. Sensorineural hearing was only mildly affected at that time. At age 14 she had severe to profound mixed hearing loss to which the bony conductance only contributed mild to moderate impairment. Her dysmorphic features, including micrognathia and hypertelorism, persisted into early adulthood and are shown in Figure 1A-B.

At age 15 years PRS-NF2 presented for the first time with a 2 week history of headache and ataxia. Brain MRI showed bilateral large acoustic masses and a left trigeminal mass (Figure 1C-D). Corpus callosum was normal. Spine MRI showed multiple extramedullary intradural lesions ranging from 1 to 7 mm in diameter (approximately 12 lesions identified from the C4-C5 level to the cauda equina; Figure 1E-F). A left-sided C7-T1 extradural lesion and a separate left-sided C7-T1 neurofibroma were also seen, and increased in size in subsequent years. No definite intramedullary lesions were identified. A holocord syrinx, likely related to a secondary Chiari malformation (inferior cerebellar tonsil herniation), was also evident. The left cerebellopontine tumor was partially-resected and identified as a schwannoma, consistent with diagnosis of NF2 (Figure 1G-H). Hence, we designated her as patient PRS-NF2. The high tumor burden, tumor progression and the patient's young age at onset of tumors fit a classification of severe NF2 [14,21-25].

**Figure 2:**
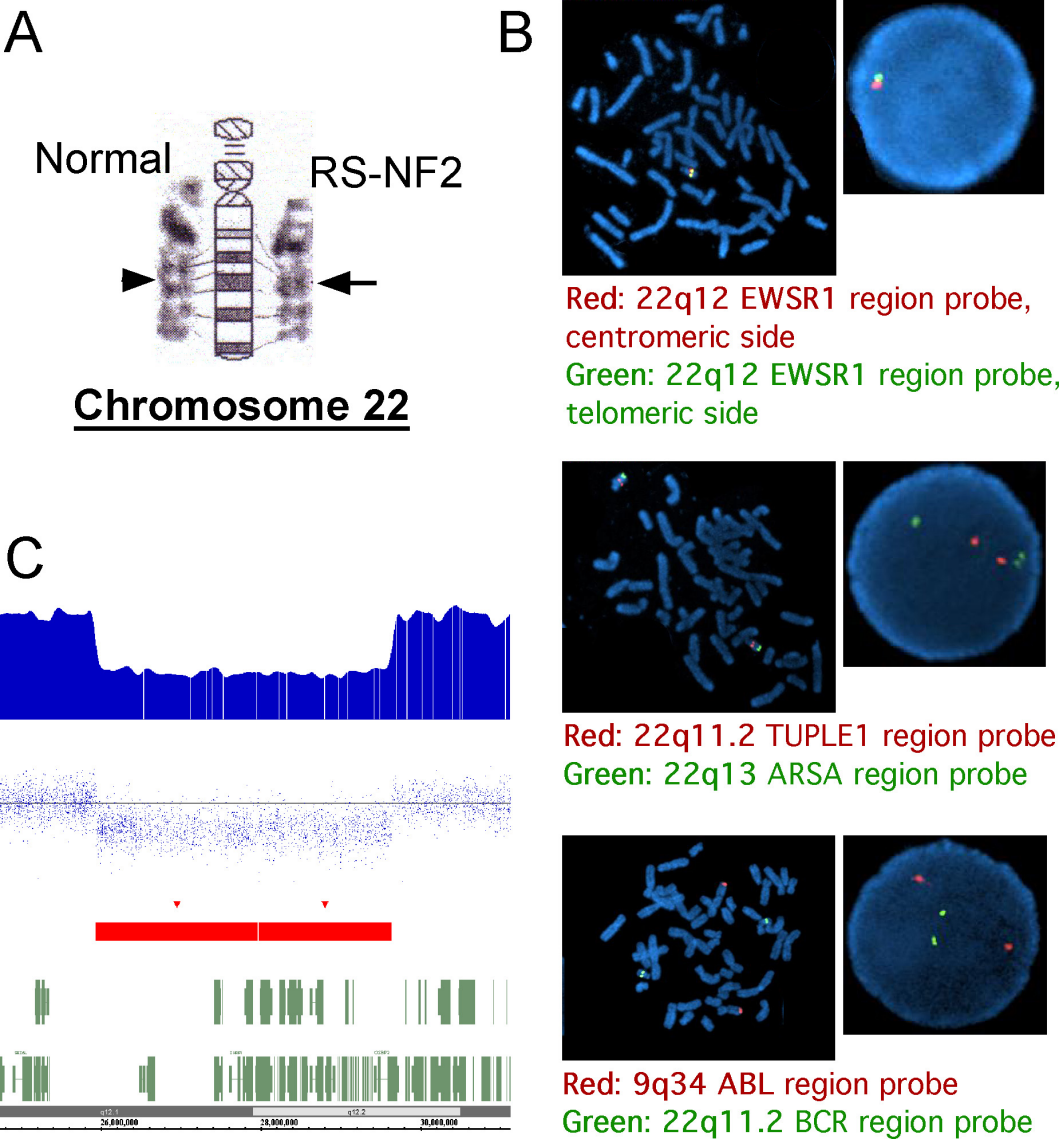
**3,693 kb deletion of 22q12.2 in patient PRS-NF2**. **A**) G-banding high resolution karyotype reveals interstitial deletion of the light band q12.2 in the long arm of chromosome 22 (arrowhead - intact normal region; arrow - deleted region). **B**) Fluorescence in situ hybridization (FISH) studies with EWSR1 (22q12.2) dual color break apart probe revealed one entire fusion signal missing indicating heterozygous deletion in 22q12.2. FISH studies with flanking probes: TUPLE1 (22q11.2) and the BCR(22q11.2)/ABL(9q34) probes showed no deletions of TUPLE or BCR. **C**) Affymetrix Genotyping Console™ (GTC) Software display of the 22q12.2 deletion identified using chromosomal microarray (CMA; Affymetrix Genome-wide Human SNP Array 6.0).

Due to the unique combination of severe NF2, facial dysmorphism and mental retardation in patient PRS-NF2, genetic evaluation was initiated. G-banding high resolution karyotype on her blood revealed 46, XX, del(22)(q12.2), indicative of an interstitial deletion of band q12.2 in the long arm of chromosome 22 (Figure 2A, arrow). FISH demonstrated a deletion of at least 1,607 kb in the 22q12.2 region (Figure 2B). Parental testing was declined. Chromosomal microarray analysis (CMA; Affymetrix SNP 6.0 array) to better delineate the mutation detected a chromosome 22q12.2 deletion of approximately 3.7 Mb (3,693 kb) spanning the *NF2 *gene, with break points at base pairs 25937541 and 29635842 (UCSC Genome Browser version hg18, release name NCBI build 36.1) (Figure 2C).

## Discussion

This is the first published report of a patient diagnosed with both PRS and NF2. Our patient's diagnosis of PRS, based on micrognathia and cleft palate, represents only part of her phenotype, which also includes microcephaly, ocular hypertelorism and mental retardation. Molecular analysis revealed a 22q12.2 deletion spanning approximately 3.7 Mb and encompassing the NF2 gene locus. The chromosomal microdeletion associated with velo-cardio-facial syndrome (22q11.2 locus) was intact in our patient. Therefore, we propose that we have identified a novel NF2-adjacent 22q12.2 microdeletion syndrome.

**Figure 3:**
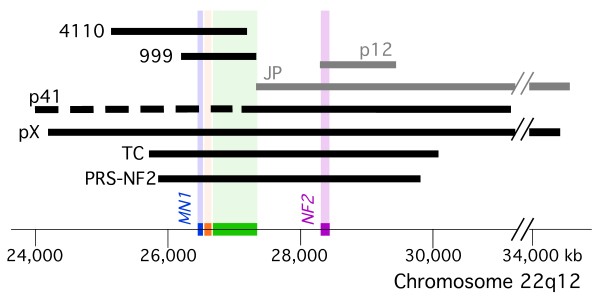
**Chromosome 22q12.2 deletions reveal loss of *MN1 *in six patients with facial dysmorphism and/or cleft palate and/or abnormal cranium**. Schema of the deletions on chromosome 22q12.2 in the eight patients described in the text and Table 1. Black bars depict the six patients with abnormal cranium and/or facial dysmorphism, and/or cleft palate. pX and TC were too young to determine whether they manifest clinical NF2. Gray bars depict the two patients without these abnormalities but with NF2. Dashed black line indicates the approximated proximal deletion in patient p41. Genes: *NF2*-purple, *TTC28*-green, *PITPNB*-orange, *MN1*-blue. Translucent vertical bars align the location of the deleted genes in relation to the chromosomal deletion found in each patient.

Review of the literature revealed only five other patients who presented with clinical features distinct from the standard diagnostic criteria of NF2 [24,26] and who had large 22q12 deletions encompassing the NF2 gene (Table 1, Figure 3). Of these five patients three are described by Bruder *et al *[19,27]. The first (JP) had a 7.4 Mb deletion of chromosome 22q12, bilateral vestibular schwannomas before the age of 25 years, mental retardation, and grand mal seizure at age 9 years, but no report of facial dysmorphism [19]. A second patient (p41) had NF2, facial dysmorphic features, mental retardation, developmental delay and a large 22q12 deletion with unknown span due to an unclear proximal deletion breakpoint [27]. The third patient (p12) had moderate NF2, mild mental retardation, and a 530 kb deletion encompassing NF2 [27]. Two other described patients, pX and TC, with large deletions at chromosome 22q12 that included *NF2 *also had severe craniofacial dysmorphism, but were too young to show stigmata of NF2 [28,29]. The first was a 10.5 month old female (pX) with facial dysmorphism including hypertelorism, mandibular hypoplasia, cleft palate, severe neurodevelopmental delay and 8 Mb deletion of chromosome 22q12.2-q12.3 [28]. Her mandibular hypoplasia and cleft palate suggest likely diagnosis of PRS. The second young patient (TC) had Toriello-Carey syndrome, including PRS with large cleft palate, micrognathia, hypertelorism, agenesis of the corpus callosum, and 6 Mb deletion overlapping much of the deletion of pX and PRS-NF2 [29]. Patient TC only walked at 22 months [29].

**Table 1:** Patient phenotypes.

	Clinical features			
**Patient**	**NF2**	**Cleft palate or craniofacial dysmorphism**	**MR**	**Other described findings**

PRS-NF2	Yes	Cleft palate Microcephaly	Yes	Micrognathia, hypertelorism, conductive hearing loss. Corpus callosum and cardiac evaluation were normal.

TC [29]	? *	Cleft palate	? **	Micrognathia, hypertelorism, absent corpus callosum, cardiac defects,

pX [28]	? *	Cleft palate	Yes	Mandibular hypoplasia, hypertelorism

p41 [27]	Yes	Facial dysmorphism	Yes	Cerebral paresis, pes cavus, peripheral facial paresis, cerebral movement disorder, cataracts

JP [19]	Yes	-	Yes	Seizure disorder

p12 [27]	Yes	-	Yes	

999 [30]	-	Microcephaly	Yes	Speech delay

4110 [30]	-	Cleft palate	Yes	Auricular pits, cataracts, deafness, hypotonia, proptosis, short phalanges

Clinical features of the eight patients described in the text [19,27-30]

* Patients pX and TC were was only 10 months old and 2 years old, respectively, at the time of the report [28,29]. While their clinical description did not include features of NF2, as would be expected at their age, their deletions did encompass the *NF2 *gene.

** Patient TC was described as having walked at age 22 months, but there is insufficient information to determine other measures of developmental and/or intellectual delay.

*NF2 *neurofibromatosis 2, *MR *mental retardation.

The young age at which PRS-NF2 was diagnosed with bilateral vestibular schwannomas (15 years), the high tumor burden that included multiple spinal tumors, and the progressive nature of some of these tumors qualify her as having a severe form of NF2 [14,23,24]. Several reports have demonstrated that nonsense/frameshift mutations are associated with a more severe phenotype than are deletions of the *NF2 *gene. This difference is thought to be due to nonsense/frameshift mutations causing a truncated protein that may have dominant negative effect, versus deletion of one copy of the *NF2 *gene, that results in decreased merlin protein expression [14,22,23,31]. Watson *et al *provided the first report on a large deletion (600-700 kb) encompassing *NF2 *that was associated with mild disease without mental retardation or facial dysmorphism [32]. Bruder *et al *described patient p12, who had a 530 kb deletion encompassing *NF2 *(partially overlapping with the patient reported by Watson *et al*) and who had moderate NF2 [27] (Figure 3, Table 1). Others support that complete deletion of the *NF2 *gene is usually associated with mild disease [33-35]. It is therefore interesting that PRS-NF2 (our patient) and patient JP [19], both of whom had large deletions encompassing the *NF2 *gene, had severe NF2 phenotype (Figure 3). Bruder *et al *hypothesized that the high severity of NF2 in JP may be due to loss of a putative second tumor suppressor gene within the 7.4 Mb deletion, that usually remained intact in patients with smaller chromosomal deletions that encompass all of *NF2 *[19]. Deletion of this putative tumor suppressor may have similarly changed a generally mild phenotype associated with complete deletion of *NF2 *to the severe phenotype seen in PRS-NF2.

The craniofacial dysmorphism in PRS-NF2 and the three other patients whose deletion overlaps her deletion (pX, p41, TC; Table 1, Figure 3) suggested a causal link between loss of one or more genes within the overlapping region of the NF2-adjacent 22q12.2 deletion and these clinical findings. To further investigate this association we searched the public database DECIPHER, which contains clinical information of individuals with chromosomal anomalies with the aim of increasing medical and scientific knowledge about these chromosomal defects [30]. DECIPHER reported two patients with chromosomal deletions in the region deleted in PRS-NF2 (Figure 3, patients 4110 and 999). Although the information is brief, there are similarities to PRS-NF2, pX, p41 and TC (Table 1): Both 4110 and 999 had mental retardation; patient 4110 had a submucous cleft palate and patient 999 had speech delay and microcephaly.

To determine genes potentially associated with the craniofacial dysmorphism and/or palatal defects in these six patients we aligned their deletions (Figure 3). The deleted area common to all six patients includes three recognized genes: *MN1*, *PITPNB *and *TTC28*, none of which has been previously linked in humans to palatal defects, calvarial abnormalities or craniofacial dysmorphism. Of these, *MN1*, initially reported as a candidate gene for sporadic meningioma and later found to be involved in acute myeloid leukemia (fusion *TEL*/*MN1*) and as an oncogene in murine hematopoiesis, was recently implicated in murine calvarial osseous development, facial membranous bone ossification, and palatal development [36-38]. Homozygous *Mn1^-/- ^*mice had calvarial bone defects with secondary cleft palate and died at birth or shortly thereafter [38,39]. Heterozygous *Mn1^+/- ^*mice showed hypoplastic membranous calvarium with incomplete penetrance of cleft palate [38], consistent with the demonstrated role of *Mn1 *in maintaining maturation and function of calvarial osteoblasts [40]. Furthermore, *Mn1 *is a transcriptional activator that regulates expression of *Tbx22 *during palatal development in mice [39]. Loss of murine *Tbx22*, which regulates intramembranous bone formation in the posterior hard palate, causes submucosal cleft palate with ankyloglossia in mice [41]. Importantly, *TBX22 *was recently also implicated in cleft palate formation in humans in that up to 5% of children with non-syndromic cleft palate have mutations in *TBX22 *[42], and *TBX22 *loss in humans is associated with X-linked cleft palate with ankyloglossia (CPX) [43]. Interestingly, the six patients we identified with *MN1 *deletion (Figure 3, Table 1) all had either craniofacial dysmorphism and/or palatal defects. Taken together, this suggests that loss of *MN1 *in these six patients may be the underlying cause for, or contributing factor to, their craniofacial dysmorphism and/or cleft palate, and supports investigation of a role for *MN1 *in human craniofacial dysmorphism and/or cleft palate.

## Conclusions

This is the first report of association between haploinsufficiency of the human *MN1 *gene (a gene associated with murine craniofacial development) and human craniofacial malformations, supporting a more in-depth investigation of a role for *MN1 *in human craniofacial development. Additionally, the overlapping phenotype of the patients described here suggests that a 22q12.2-contiguous gene deletion syndrome should be considered in individuals with hearing loss, developmental deficits and craniofacial dysmorphism that includes Pierre-Robin Sequence or isolated micrognathia with or without cleft palate. As microarray testing replaces chromosome and FISH testing, large deletions such as the one found in our patient are more likely to be found when interrogating cases of PRS. This may allow earlier diagnosis of similar contiguous gene deletions and provide for earlier surveillance of serious clinical disorders such as NF2.

## Consent

Written informed permission for use and disclosure of this patient's protected health information for research purposes and permission to obtain and release the accompanying photographs were obtained from the patient's parents. Copies of the consents are available for review by the Editor-in-Chief of this journal.

## Abbreviations

MN1: Meningioma1; NF2: Neurofibromatosis type 2; PRS: Pierre-Robin Sequence.

## Competing interests

The authors declare that they have no competing interests.

## Authors' contributions

All authors reviewed the manuscript critically for its content, revised and edited it, and approved the final version. Additionally, specific author contributions are as follows: TBD coordinated data acquisition, analyzed and interpreted the data and drafted the manuscript. PA S-L participated in analysis and interpretation of the molecular data, search of the DECIPHER database and drafting of the manuscript. LMR and MDK acquired and interpreted the clinical data and drafted the manuscript. SW performed and interpreted the karyotype and FISH analysis. AP analyzed and interpreted the MRI imaging data. HS analyzed and interpreted the pathological slides. AE-E conceptualized, designed and coordinated the study, acquired and interpreted data, drafted the manuscript and finalized it. All authors read and approved the final manuscript.

## Pre-publication history

The pre-publication history for this paper can be accessed here:

http://www.biomedcentral.com/1471-2350/13/19/prepub
